# Modified transesophageal echocardiography of the dissected thoracic aorta; a novel diagnostic approach

**DOI:** 10.1186/s12947-016-0071-6

**Published:** 2016-08-03

**Authors:** Wouter W. Jansen Klomp, Linda M. Peelen, George J. Brandon Bravo Bruinsma, Arnoud W. J. van’t Hof, Jan G. Grandjean, Arno P. Nierich

**Affiliations:** 1 Department of Cardiology, V2.2, ISALA, Dokter van Heesweg 2, 8025AB Zwolle, The Netherlands; 2 Department of Clinical Epidemiology, Julius Center for Health Sciences and Primary Care, University Medical Center Utrecht, P.O. Box 85500, 3508 GA Utrecht, The Netherlands; 3 Department of Anesthesiology, University Medical Center Utrecht, P.O. Box 85500, 3508 GA Utrecht, The Netherlands; 4 Department of Cardiothoracic Surgery, ISALA, Dokter van Heesweg 2, 8025AB Zwolle, The Netherlands; 5 MIRA Institute for Biomedical Technology and Technical Medicine, University of Twente, P.O. box 217, 7500 AE Enschede, The Netherlands; 6 Department of (Thoracic) Anaesthesia and Intensive Care, ISALA, Dokter van Heesweg 2, 8025AB Zwolle, The Netherlands

**Keywords:** Transesophageal echocardiography, Aortic dissection, Cardiothoracic surgery, Cerebral monitoring

## Abstract

**Background:**

Transesophageal echocardiography (TEE) is a key diagnostic modality in patients with acute aortic dissection, yet its sensitivity is limited by a “blind-spot” caused by air in the trachea. After placement of a fluid-filled balloon in the trachea visualization of the thoracic aorta becomes possible. This method, modified TEE, has been shown to be an accurate test for the diagnosis of upper aortic atherosclerosis. In this study we discuss how we use modified TEE for the diagnosis and management of patients with (suspected) acute aortic dissection.

**Novel diagnostic approach of the dissected aorta:**

Modified TEE provides the possibility to obtain a complete echocardiographic overview of the thoracic aorta and its branching vessels with anatomical and functional information. It is a bedside test, and can thus be applied in hemodynamic instable patients who cannot undergo computed tomography. Visualization of the aortic arch allows differentiation between Stanford type A and B dissections and visualization of the proximal cerebral vessels enables a timely identification of impaired cerebral perfusion.

During surgery modified TEE can be applied to identify the true lumen for cannulation, and to assure that the true lumen is perfused. Also, the innominate- and carotid arteries can be assessed for structural integrity and adequate perfusion during multiple phases of the surgical repair.

**Conclusions:**

Modified TEE can reveal the “blind-spot” of conventional TEE. In patients with (suspected) aortic dissection it is thus possible to obtain a complete echocardiographic overview of the thoracic aorta and its branches. This is of specific merit in hemodynamically unstable patients who cannot undergo CT. Modified TEE can guide also guide the surgical management and monitor perfusion of the cerebral arteries.

**Electronic supplementary material:**

The online version of this article (doi:10.1186/s12947-016-0071-6) contains supplementary material, which is available to authorized users.

## Background

Acute aortic dissection (AD) is a life-threatening condition, which requires a prompt diagnosis to prevent morbidity and mortality. This necessitates sensitive and conclusive diagnostic tests. Transthoracic echocardiography is often the first test used since it is directly available, may reveal a proximal dissection, and can detect pericardial and pleural effusion [1, 2]. However, the focused screening for AD requires tests with a superior diagnostic accuracy, i.e. computed tomography (CT), magnetic resonance imaging (MRI) or transesophageal echocardiography (TEE) [3, 4].

Guidelines recommend the use of CT or MRI as the primary test in patients who are hemodynamically stable [5]. Both modalities can accurately visualize an intimal tear, and complications can be detected, including aneurysmal widening of the aorta, pericardial and pleural effusion and involvement of coronary or distal arteries [3–6]. A major limitation of both tests is the need to move patients out of the acute care environment. Moreover, CT is lacking the possibility of functional imaging, while the use of MRI is limited by its longer imaging-time and lower availability.

In hemodynamically instable patients, TEE is recommended as the primary test because it can be performed in the emergency care department or operating room [3–5]. Transesophageal echocardiography has the advantage of providing a quick overview of the heart and aorta with both anatomical and functional information. The diagnostic accuracy of TEE is considered inferior however compared to CT and MRI, mainly because of the impaired visualization of the distal ascending aorta and aortic arch caused by the interposition of the air-filled trachea [3, 4, 7, 8]. Indeed, limited dissections can be missed due to this so-called “blind-spot” [9].

**Fig. 1:**
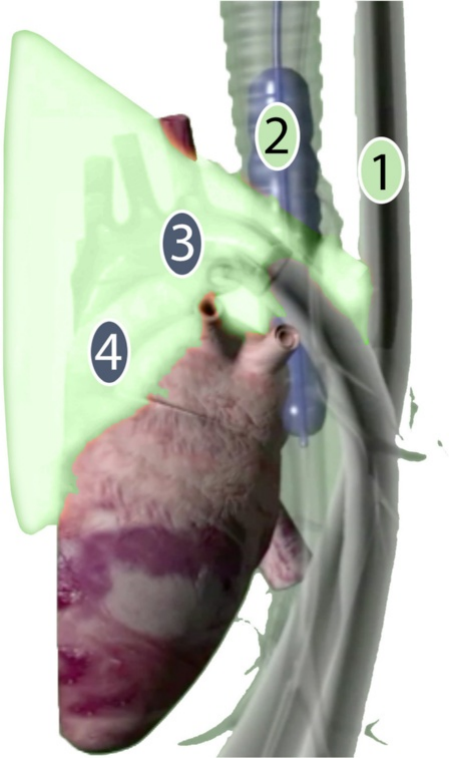
Schematic overview of modified transesophageal echocardiography. 1. Esophagus with TEE probe; 2. Trachea and left main bronchus with inflated endotracheal “A-View” balloon creating an echocardiographic window to the aortic arch; 3. Distal ascending aorta, aortic arch and branching vessels; 4. Pulmonary artery

The diagnostic accuracy of conventional TEE can be improved using a modification which enables the visualization of the distal ascending aorta, aortic arch and its branches through a fluid-filled balloon placed in the trachea and left main bronchus (Fig. 1) [10]. The original aim of “modified TEE” was to improve the diagnosis of aortic atherosclerosis during cardiothoracic surgery and thereby reduce the incidence of emboli-related complications. Modified TEE is indeed a sensitive test for the diagnosis of atherosclerosis of the distal ascending aorta [10–12], with a superior diagnostic accuracy compared to conventional TEE [13].

We also routinely use modified TEE in patients with (suspected) AD. Most importantly, modified TEE provides the possibility to obtain a complete echocardiographic overview of the thoracic aorta and its branches in patients who cannot undergo CT. Second, modified TEE can guide essential steps in the surgical repair of the dissected aorta. In this study we discuss how we use modified TEE to aid the diagnosis and management of AD patients.

### Technique of modified TEE

**Fig. 2:**
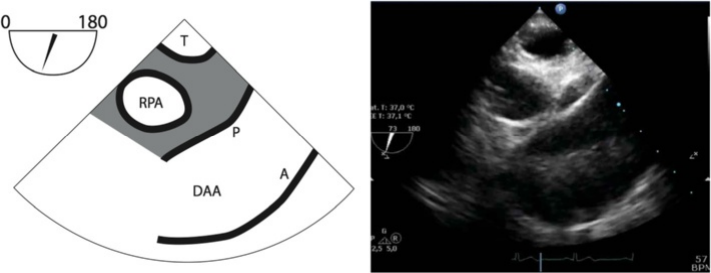
Distal ascending aorta long-axis view with modified TEE showing a normal aorta. T = Trachea with inflated endotracheal balloon. RPA = Right Pulmonary Artery, DAA = Distal Ascending Aorta, P = Posterior wall, A = Anterior wall

**Fig. 3:**
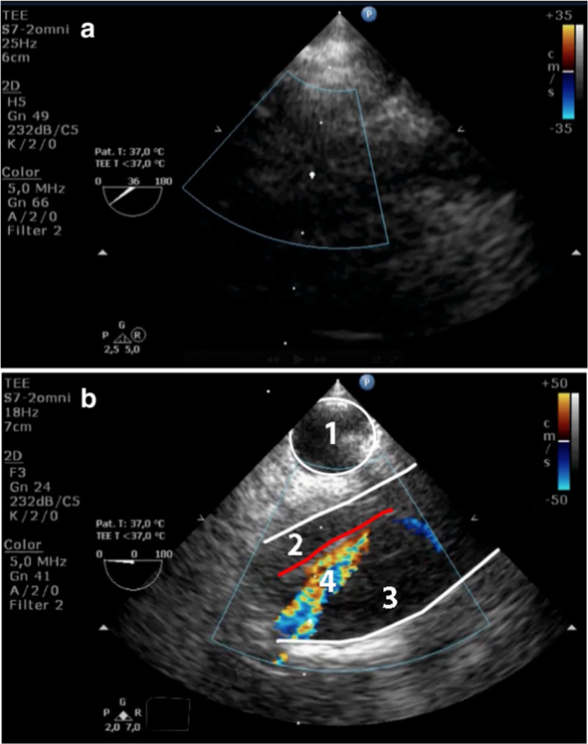
Visualization of the distal ascending aorta (DAA) during elective aortic root replacement in a 43-year old woman with Marfan syndrome; the bispectral index indicated a compromised cerebral perfusion. Panel (**a**) Conventional TEE with inadequate visualization of the cannulation site due to the “blind-spot”. Panel (**b**) Same view (DAA, long-axis) after inflation of the endotracheal balloon, showing a limited iatrogenic dissection (red line) with extracorporeal perfusion of the false lumen. 1. Trachea with inflated endotracheal balloon; 2. True lumen; 3. False lumen; 4. Perfusion jet

A conventional TEE examination of the heart and aorta is performed following the prevailing guidelines [14]. In the setting of suspected AD, it is recommended to perform TEE under general anaesthesia as the introduction of the probe may cause an inadvertent increase of the blood pressure in conscious patients [4], which is associated with a higher likelihood of rupture of the adventitial layer [15]. The endotracheal-tube should be placed directly distal from the vocal chords to ensure enough space for the positioning of the tracheal balloon. Pre-oxygenation permits for an apnoeic period of 2–3 min in which imaging with modified TEE can be performed safely. During cardiac bypass or deep hypothermic arrest, modified TEE can be used continuously. After preparation of the specially designed “A-View” catheter [12], the ventilator is disconnected, and the A-View catheter is introduced and positioned in the trachea and left main bronchus. For a correct positioning, the 24 cm markers on the endotracheal tube and catheter should line-up; a correct positioning of the catheter can also be checked using TEE. Inflation of the balloon with 20–50 ml of saline should give an echocardiographic window to the “blind-spot” (Figs. 2 and 3).

Modified TEE is compatible with additional modalities including color-Doppler and 3D imaging. Contraindications are similar as for conventional TEE, with the addition of tracheomalacia (e.g. in connective tissue diseases) and tracheal stenosis which may predispose to complications related to the balloon inflation.

### Examination in (suspected) aortic dissection

In the majority of patients CT is used as the primary diagnostic test for AD, with (modified) TEE as a secondary test to assess the functional consequences of an aortic dissection. Therefore, modified TEE is usually performed in the operating room before sternotomy, although imaging can also be performed in the emergency room. First, TEE is used to identify aortic dilatation, aortic valve insufficiency, and pericardial or pleural effusion. Then, the proximal ascending aorta and descending aorta are screened for a dissection, followed by an attempt the visualize the upper thoracic aorta and its branches. Usually no echocardiographic window can be obtained however, and the procedure will continue with modified TEE.

#### Distal ascending aorta view

**Fig. 4:**
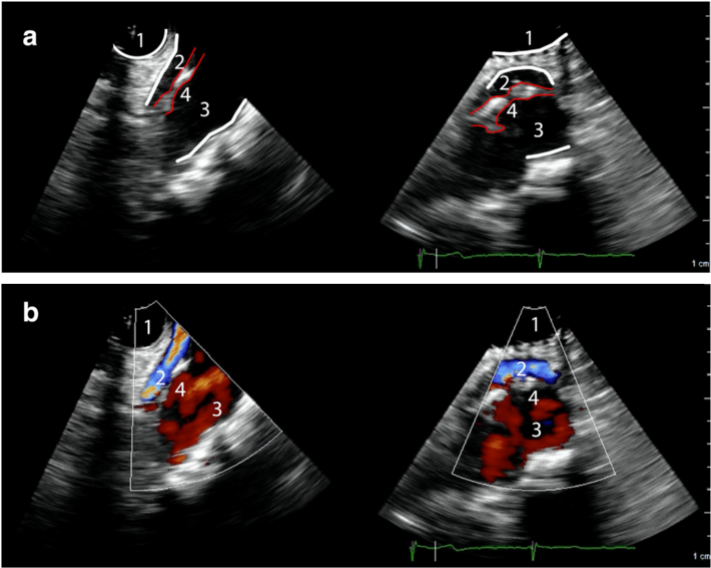
Panel (**a**) Modified TEE long- and short axis (X-plane) view of the distal ascending aorta (DAA), showing an intimal tear. Panel (**b**) Same image with color-Doppler showing differential flow in the true- and false lumen. 1 = Trachea with inflated endotracheal balloon; 2 = True lumen; 3 = False lumen; 4 = Intimal tear

From the conventional mid-esophageal ascending aortic view, the short-axis distal ascending aortic view should be obtained after retraction of the probe until a depth of 25 to 30 cm from the incisors, with the multiplane angle adjusted to 30° (Fig. 4; Additional file 1 and 2) [16]. A long-axis view can be obtained by adjusting the multiplane angle to 70–120°. As in any echo-based test, reverberation artefacts may mimic an intimal tear [17]. Suspicion of an intimal tear should therefore always be confirmed in multiple views, and color-Doppler should be used to reveal differential flow in the true and false lumen, if possible to show flow through an entry or exit tear, and reveal (bi) directional flow. Additional signs indicative for a false lumen include spontaneous contrast, partial or complete thrombosis and diastolic expansion [3].

#### Aortic arch view

**Fig. 5:**
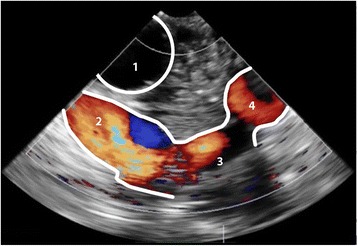
Modified TEE aortic arch short-axis view in a patient with a normal aortic arch and flow pattern. 1 = Trachea with inflated endotracheal balloon; 2 = Innominate artery; 3 = Aortic arch; 4 = Left carotid artery

**Fig. 6:**
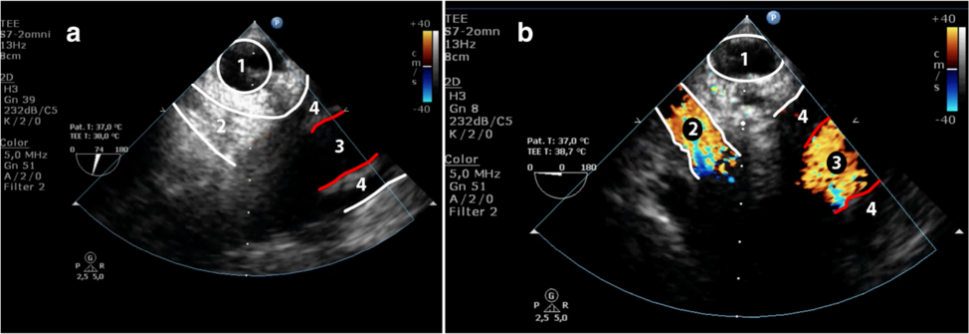
Modified TEE aortic arch short axis view. Panel (**a**) Plain 2D image showing a dissection starting in the proximal aortic arch, which continued distally into the descending aorta. The intimal tear did not progress into the innominate artery. Panel (**b**) Similar image with color-Doppler showing flow through the true aortic lumen, and a normal perfusion of the innominate artery. 1 = Trachea with inflated endotracheal balloon; 2 = Innominate artery; 3 = True lumen; 4 = False lumen; red lines delineate the intimal tear

After the TEE probe is retracted another 2 to 5 cm with the multiplane angle at 70°, the aortic arch is visualized (Fig. 5 and Additional file 3). Adjustment of the angle to 0° will reveal a short-axis view of the aortic arch with the innominate artery and the left subclavian artery on the right and left side of the image respectively. Again, an intimal tear should be confirmed in two directions, preferably with additional signs differentiating a false and true lumen (Fig. 6). This view may be of particular importance to differentiate Stanford type A and type B dissection.

#### Aortic branch vessels view

**Fig. 7:**
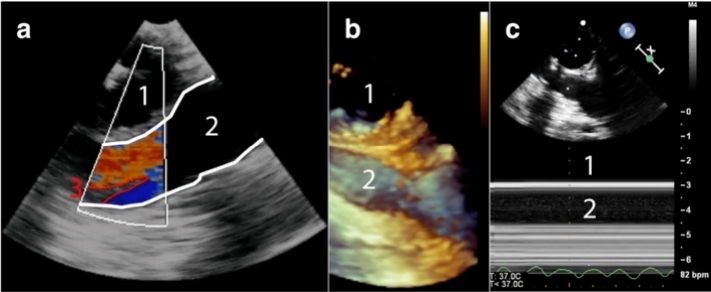
Modified TEE showing the innominate artery with three different modalities. Panel (**a**) 2D-image showing an intimal tear and differential flow. Panel (**b**) 3D-image in a different patient without dissection. Panel (**c**) M-Mode image of the same patient without dissection. 1 = Trachea with inflated endotracheal balloon; 2 = Innominate artery; 3 = Intimal tear

Finally, the proximal part of the innominate artery (Fig. 7; Additional file 4, Fig 8; Additional file 5 and 6), and the left carotid- and subclavian artery are visualized by further retracting the TEE probe. Navigation between the vessels is achieved by rotation of the TEE probe both to the left and right.

**Fig. 8:**
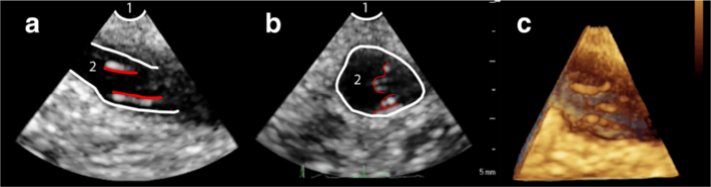
Modified TEE image of a patient with Stanford type A aortic dissection with progression into the innominate artery, visualized in a 2D long-axis (Panel **a**) and short-axis view (Panel **b**), and in a 3D long-axis view (panel **c**). 1. Trachea with inflated endotracheal balloon 2. Innominate artery; red lines delineating the intimal tear

### Perioperative monitoring

During surgery for AD, modified TEE is used to guide the surgical management and to support cerebral monitoring. Specific attention is given to the perfusion of the branching vessels (i.e. the brachiocephalic- and carotid artery), which can be impaired because of a continuing dissection, obstruction by an intimal tear, or compression by an extravascular hematoma. The early detection of impaired cerebral perfusion allows for timely changes in the management, e.g. direct initiation of deep-hypothermic arrest, maintenance of a higher blood pressure and application of additional cerebral monitoring tools, e.g. bispectral index and near-infrared spectroscopy.

**Fig. 9:**
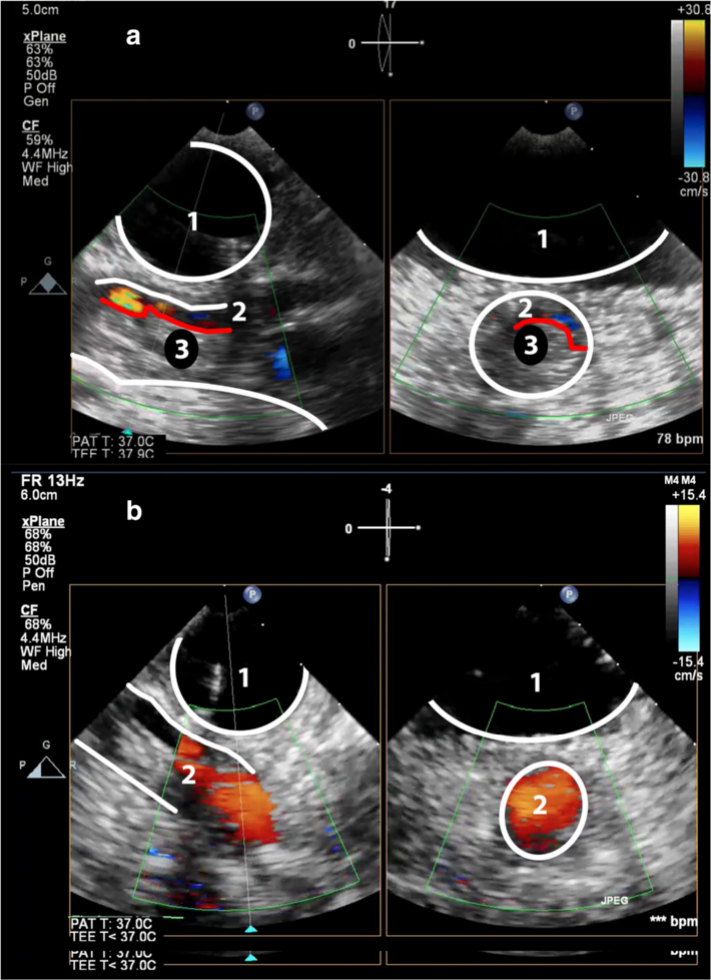
Modified TEE long- and short axis (X-plane) images of the innominate artery. Panel (**a**) Image before surgery, showing propagation of an intimal tear into the innominate artery with almost complete obstruction of the vessel with limited perfusion of the true lumen. Panel (**b**) Image after surgery showing a normal flow pattern during extracorporeal circulation; artefact in the long-axis view, which does not indicate a persistent dissection. 1 = Trachea with inflated endotracheal balloon; 2 = Innominate artery

During surgery we use modified TEE to guide aortic cannulation and to confirm that the true lumen is perfused during CPB. If right axillary cannulation is considered, the structural integrity of the innominate artery is inspected first. We check the flow in the right subclavian artery and right- and left carotid artery again after the extracorporeal circulation has started, and finally after surgical repair to confirm an adequate surgical result (Fig. 9; Additional files 7 and 8).

### Limitations of modified TEE

Modified TEE has some limitations. First, experience with conventional TEE is a prerequisite and additional training should be considered since a learning curve exists. Second, the spatial resolution can be lower than in conventional TEE because of some scattering by the balloon. Finally, we already addressed the procedures to prevent false positive results. Despite these measures we are aware of one patient (female, 43 years) who underwent sternotomy because of a false positive finding with modified TEE. This patient was referred for surgery based on the suspicion of an AD on CT. During preoperative visualization of the thoracic aorta with modified TEE no intimal tear was confirmed, but a structure outside the aorta appeared as an extramural hematoma of the aortic arch. However, during intraoperative inspection this structure was revealed to be thymus tissue.

## Conclusions

Modified TEE can reveal the “blind-spot” of conventional TEE. In patients with suspected acute aortic dissection it is thus possible to obtain a complete echocardiographic overview of the thoracic aorta and its branches with both anatomical and functional information. This may be specifically useful in hemodynamically unstable patients who cannot undergo CT. During surgery for aortic dissection, modified TEE can guide the surgical management and monitor perfusion of the cerebral arteries.

## Abbreviations

AD, acute aortic dissection; CT, computed tomography; TEE, transesophageal echocardiography

## Additional files

Additional file 1: Modified TEE long- and short axis (X-plane) view of the distal ascending aorta (DAA), showing an intimal tear. (MP4 2793 kb)

Additional file 2: Modified TEE long- and short axis (X-plane) view of the distal ascending aorta (DAA) with color-Doppler showing differential flow in the true- and false lumen. (MP4 2737 kb)

Additional file 3: Modified TEE aortic arch short-axis view in a patient with a normal aortic arch and flow pattern. (MP4 589 kb)

Additional file 4: Modified TEE showing the innominate artery with an intimal tear and differential flow. (MP4 2327 kb)

Additional file 5: Modified TEE video of a patient with Stanford type A aortic dissection with progression into the innominate artery, visualized in a 2D long-axis and short-axis view. (MP4 1524 kb)

Additional file 6: Modified TEE video of a patient with Stanford type A aortic dissection with progression into the innominate artery, visualized in a 3D long-axis view. (MP4 2641 kb)

Additional file 7: Modified TEE long- and short axis (X-plane) videos of the innominate artery before surgery, showing propagation of an intimal tear into the innominate artery with almost complete obstruction of the vessel with limited perfusion of the true lumen. (MP4 491 kb)

Additional file 8: Modified TEE long- and short axis (X-plane) videos of the innominate artery after surgery showing a normal flow pattern during extracorporeal circulation; artefact in the long-axis view, which does not indicate a persistent dissection. (MP4 440 kb)
