# Erratum to: ‘Modified transesophageal echocardiography of the dissected thoracic aorta; a novel diagnostic approach’

**DOI:** 10.1186/s12947-016-0082-3

**Published:** 2016-09-08

**Authors:** Wouter W. Jansen Klomp, Linda M. Peelen, George J. Brandon Bravo Bruinsma, Arnoud W. J. van’t Hof, Jan G. Grandjean, Arno P. Nierich

**Affiliations:** 1 Department of Cardiology, V2.2, ISALA, Zwolle, The Netherlands; 2 Department of Clinical Epidemiology, Julius Center for Health Sciences and Primary Care, University Medical Center Utrecht, Utrecht, The Netherlands; 3 Department of Anesthesiology, University Medical Center Utrecht, Utrecht, The Netherlands; 4 Department of Cardiothoracic Surgery, ISALA, Zwolle, The Netherlands; 5 MIRA Institute for Biomedical Technology and Technical Medicine, University of Twente, Enschede, The Netherlands; 6 Department of (Thoracic) Anaesthesia and Intensive Care, ISALA, Zwolle, The Netherlands

## Erratum

Unfortunately, the original version of this article [1] contained an error. The competing interests section was published with an error.

“WJK received financial support for an E-learning course and congress presentation from Medical2Market B.V. AN holds stock in Medical2Market B.V., Zwolle, the Netherlands.”

should be:

“WJK received financial support for an E-learning course and congress presentation from Stroke2prevent B.V. and AN holds stock in Stroke2prevent B.V., Zwolle, the Netherlands.”
